# A stitch in time saves nine: external quality assessment rounds demonstrate improved quality of biomarker analysis in lung cancer

**DOI:** 10.18632/oncotarget.24980

**Published:** 2018-04-17

**Authors:** Cleo Keppens, Véronique Tack, Nils ‘t Hart, Lien Tembuyser, Ales Ryska, Patrick Pauwels, Karen Zwaenepoel, Ed Schuuring, Florian Cabillic, Luigi Tornillo, Arne Warth, Wilko Weichert, Elisabeth Dequeker, for the EQA assessors expert group

**Affiliations:** ^1^ University of Leuven, Department of Public Health and Primary Care, Biomedical Quality Assurance Research Unit, Leuven, Belgium; ^2^ University Medical Center Groningen, Department of Pathology, Groningen, The Netherlands; ^3^ Charles University Medical Faculty and University Hospital, Department of Pathology, Hradec Kralove, Czech Republic; ^4^ Center for Oncologic Research (CORE), University of Antwerp, Antwerp, Belgium; ^5^ University Hospital Antwerp, Department of Pathology, Edegem, Belgium; ^6^ Cytogenetics and Cellular Biology Department, CHU de Rennes, Rennes, France; ^7^ INSERM, INRA, Université Rennes 1, Université Bretagne Loire, Nutrition Metabolisms and Cancer, Rennes, France; ^8^ University of Basel, Basel, Switzerland; ^9^ GILAB AG, Allschwil, Switzerland; ^10^ University Hospital Heidelberg, Heidelberg, Germany; ^11^ Technical University Munich (TUM), Munich, Germany; ^12^ The EQA assessors expert group

**Keywords:** non-small cell lung cancer, external quality assessment, molecular pathology, biomarker analysis, targeted therapy

## Abstract

Biomarker analysis has become routine practice in the treatment of non-small cell lung cancer (NSCLC). To ensure high quality testing, participation to external quality assessment (EQA) schemes is essential. This article provides a longitudinal overview of the EQA performance for *EGFR*, *ALK*, and *ROS1* analyses in NSCLC between 2012 and 2015.

The four scheme years were organized by the European Society of Pathology according to the ISO 17043 standard. Participants were asked to analyze the provided tissue using their routine procedures.

Analysis scores improved for individual laboratories upon participation to more EQA schemes, except for ROS1 immunohistochemistry (IHC). For *EGFR* analysis, scheme error rates were 18.8%, 14.1% and 7.5% in 2013, 2014 and 2015 respectively. For *ALK* testing, error rates decreased between 2012 and 2015 by 5.2%, 3.2% and 11.8% for the fluorescence *in situ* hybridization (FISH), FISH digital, and IHC subschemes, respectively. In contrast, for *ROS1* error rates increased between 2014 and 2015 for FISH and IHC by 3.2% and 9.3%. Technical failures decreased over the years for all three markers.

Results show that EQA contributes to an ameliorated performance for most predictive biomarkers in NSCLC. Room for improvement is still present, especially for *ROS1* analysis.

## INTRODUCTION

Over the last decades, personalized health care has become routine practice in the treatment of patients with non-small cell lung cancer (NSCLC) and several clinically important companion diagnostics have been implemented [1]. More specifically, testing for epidermal growth factor receptor (*EGFR*) variants in exons 18 to 21 is required by the Food and Drug Administration (FDA) and European Medicines Agency (EMA) prior to treatment of patients with EGFR tyrosine kinase inhibitors (TKIs) [2, 3]. Similarly, rearrangements of the anaplastic lymphoma kinase (*ALK*) and ROS proto-oncogene 1 tyrosine-protein kinase (*ROS1*) genes can predict the treatment outcome of TKIs as well [2, 3]. While the *ALK* gene has been included in the drug label of crizotinib since 2011, testing of *ROS1* rearrangements for treatment of patients with NSCLC has recently been added to the label of crizotinib by the FDA (March 2016) and by the EMA (September 2016) for first line therapy [4–6].

In 2012, lung cancer displayed the highest incidence of cancer onset and mortality worldwide [7], with 80% of cases due to NSCLC. High quality molecular testing in NSCLC is of utmost importance to prevent false-positive or false-negative results that could diminish patient prognosis or evoke unnecessary adverse treatment effects [7]. In addition, emerging techniques for molecular tumor analyses, such as next-generation sequencing (NGS) and the identification of novel molecular targets, pose new challenges. A lack of quality control could compromise correct test results [8–10]. As a result, laboratories are challenged to implement and also to maintain accurate test procedures to offer reliable results within an acceptable timeframe. Indeed, an increased error rate has been reported in 2013 following the introduction of full *RAS* testing for colorectal cancer [11]. Therefore, adequate training is necessary to avoid potential pitfalls, before a predictive molecular test is implemented in routine practice.

Participation to external quality assessment (EQA) schemes, also called proficiency testing, allows to monitor laboratory performance, to compare them on an inter-laboratory level and to provide individual feedback. The aim of EQA schemes is to educate and support diagnostic laboratories to reach an accurate quality level of test results [7, 12]. In addition, EQA participation is an integral part of the quality framework of diagnostic laboratories, required by the International Organization for Standardization (ISO 15189) [15] and the Clinical Laboratory Improvement Amendments [13].

In 2012 and 2013, the European Society of Pathology (ESP) introduced two pilot EQA schemes to evaluate the performance of *ALK* analysis in NSCLC [17]. In 2013, results showed a decreased error rate from 7.3% to 5.2% for *ALK* fluorescence *in situ* hybridization (FISH) and from 13.0% to 8.2% for ALK immunohistochemistry (IHC) as compared to 2012. In spite of these improvements, error rates and enumeration practices of the number of FISH nuclei, could still be ameliorated [17]. Results from other European EQA providers reported similar error rates for biomarker analysis in NSCLC, emphasizing the need for continued education [14–18].

The aim of this article is to analyze the results of four subsequent ESP Lung EQA schemes between 2012 and 2015, as well as the laboratory improvement on a longitudinal level. A comparison will be made between three predictive biomarkers in NSCLC: *EGFR, ALK* and *ROS1*.

## RESULTS

Over four years, 221 unique laboratories participated to the *EGFR* scheme, 243 to the *ALK* scheme (FISH and/or IHC) and 98 to the *ROS1* scheme (FISH and/or IHC). In 2014, 31, 63 and 117 laboratories registered for three, two or one of these markers respectively. In 2015, 35 laboratories participated to the schemes for all three markers, 62 laboratories participated to the schemes for two of three markers and 99 participants to only one of the markers. More than half of the laboratories were affiliated to a university (hospital) or were involved in both diagnostics and research (*EGFR* 56.6%, *ALK*, 56.8%, and *ROS1* 62.2%). Less than a third of the participants were affiliated to a general or private hospital (*EGFR* 24.4%, *ALK* 28.0%, and *ROS1* 21.4%) and a minority were private laboratories (*EGFR* 16.7%, *ALK* 13.2%, and *ROS1* 13.3%) or industrial ones (*EGFR* 2.3%, *ALK* 2.1%, and *ROS1* 3.1%). Only a third of the institutes were accredited according to an international standard or national equivalent during their most recent EQA participation (*EGFR* 32.1%, *ALK* 32.5%, and *ROS1* 38.8%).

The individual performance of the laboratories improved when they participated to more EQA scheme years. Namely, their average performance score improved for two, three or four subsequent participations. (Table 1). Only for the ROS1 IHC subscheme, the average performance score did not improve after repeated participation, unless one challenging positive ROS1 IHC case was not taken into account.

**Table 1:** Average performance score according to the number of years that a laboratory participated to the EQA scheme

EQA participation	Average performance score									
	*EGFR*		*ALK* FISH		ALK IHC		*ROS1* FISH		ROS1 IHC	
	N=	%	N=	%	N=	%	N=	%	N=	%
1^st^	222	78.8	184	90.0	125	91.0	90	86.3	41	92.1
2^nd^	99	90.0	113	90.9	74	94.9	34	94.9	21	86.7 (97.1*)
3^rd^	48	91.0	60	93.7	51	96.7	NA	NA	NA	NA
4^th^	NA	NA	24	95.4	18	100.0	NA	NA	NA	NA

The scheme period included in the analysis, ranges from 2012 to 2015. The number represents the number of laboratories who participated to only one, two, three, or four years of EQA for that specific marker or subscheme. *Average score with the exclusion of a challenging positive ROS1 IHC case in 2015. Abbreviations: *ALK*, anaplastic lymphoma kinase; *EGFR*, epidermal growth factor receptor; EQA, external quality assessment; FISH, fluorescence *in situ* hybridization; IHC, immunohistochemistry; *ROS1*, proto-oncogene 1 tyrosine-protein kinase; N, number of laboratories; NA, not applicable because no EQA scheme was organized in this year.

**Figure 1:**
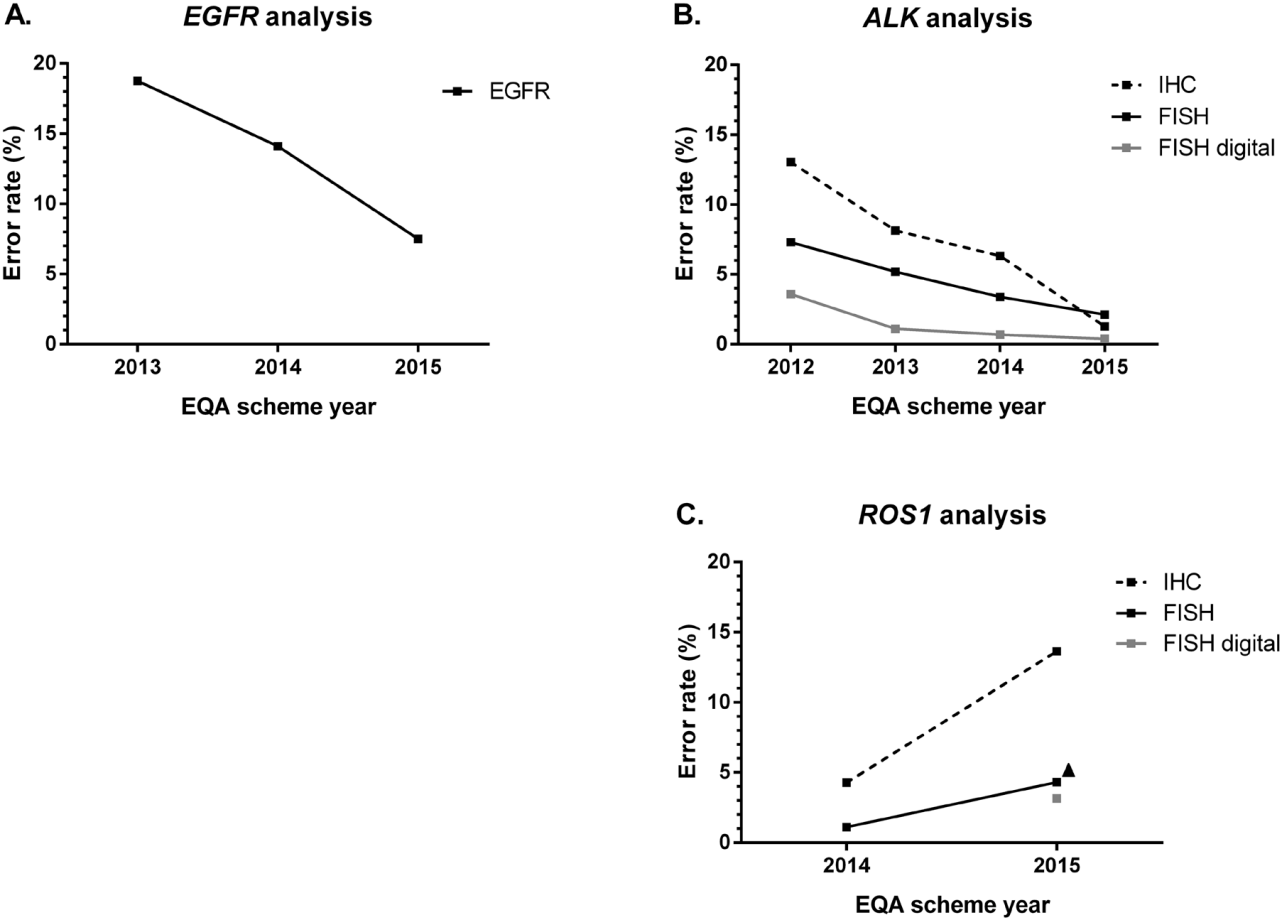
Error rates (in percentage) across EQA scheme years Errors rates include false-positive and false-negative results for *EGFR*, *ALK* and *ROS1* and wrong variants for *EGFR* on the total number of analyzable samples. Educational samples, samples for which less than 50 nuclei were enumerated or for which a technical failure occurred, were not taken into account. **(A)** Error rates for *EGFR* variant analysis. N = 428 (2013), 1296 (2014), 1026 (2015). **(B)** Error rates for *ALK* analysis in different subschemes. IHC: n = 232 (2012), 576 (2013), 864 (2014), 475 (2015); FISH: n = 269 (2012), 500 (2013), 833 (2014), 520 (2015); FISH digital: n = 260 (2012), 424 (2013), 291 (2014), 522 (2015). **(C)** Error rates for *ROS1* analysis in different subschemes. IHC: n = 310 (2014), 155 (2015); FISH: n = 372 (2014), 325 (2015); FISH digital: n = 259 (2015). *ALK*, anaplastic lymphoma kinase; *EGFR*, epidermal growth factor receptor; EQA, external quality assessment; FISH, fluorescence *in-situ* hybridization; IHC, immunohistochemistry; N, number of participants; *ROS1*, proto-oncogene 1 tyrosine-protein kinase.

**Figure 2:**
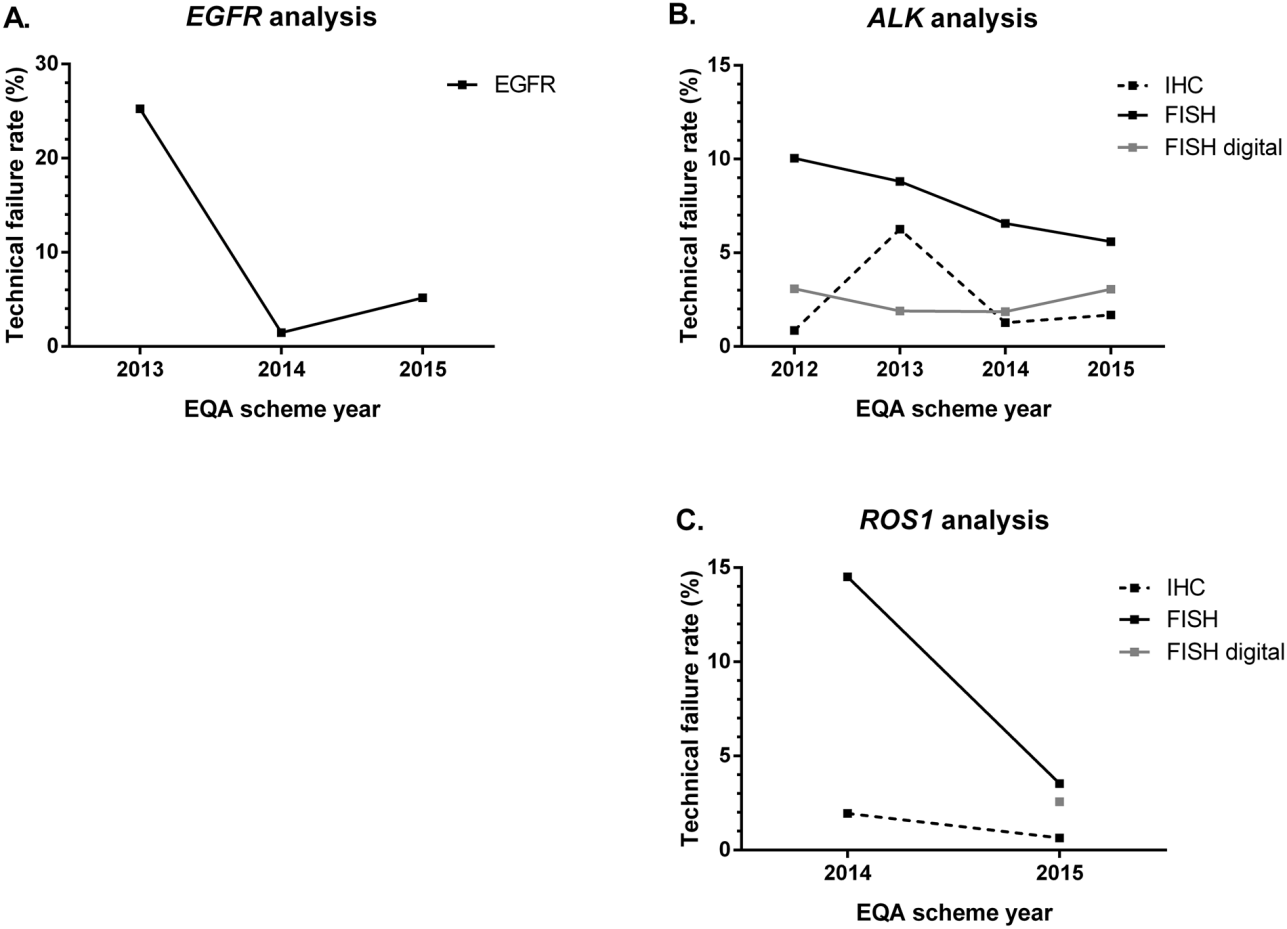
Technical failure rates (in percentage) across EQA scheme years Educational samples were not taken into account for calculation of the percentages. **(A)** Error rates for *EGFR* variant analysis. N= 428 (2013), 1296 (2014), 1026 (2015). **(B)** Error rates for *ALK* analysis in different subschemes. FISH: n = 269 (2012), 500 (2013), 928 (2014), 555 (2015); FISH digital: n = 260 (2012), 424 (2013), 324 (2014), 555 (2015); IHC: n = 232 (2012), 576 (2013), 864 (2014), 475 (2015). **(C)** Error rates for *ROS1* analysis in different subschemes. FISH: n = 448 (2014), 340 (2015); FISH digital: n = 272 (2015); IHC: n = 310 (2014), 155 (2015). *ALK*, anaplastic lymphoma kinase; *EGFR*, epidermal growth factor receptor; EQA, external quality assessment; FISH, fluorescence *in-situ* hybridization; IHC, immunohistochemistry; N, Number of participants; *ROS1*, proto-oncogene 1 tyrosine-protein kinase.

Error rates and the rates of technical failures during the EQA rounds for *EGFR, ALK* and *ROS1* analysis are summarized in Figure 1 and Figure 2, respectively. For *EGFR* analysis, there is a decrease in error rate between 2013 and 2015 from 18.8% towards 14.1% and 7.5% (Figure 1A). Erroneous results were mainly caused by missing the c.2369C>T p.(Thr790Met) variant. There was no correlation between false-negative results for this variant and the use of a specific methodology. Exclusion of these samples yielded error rates of 18.0%, 14.5% and 6.4% in the 2013, 2014 and 2015 *EGFR* schemes, respectively. The estimation of the percentage of neoplastic cells for *EGFR* variant analysis displayed a high inter-observer variability, with an average standard deviation of 19.8% (n=142) and 21.1% (n=114) in 2014 and 2015 for four and nine samples, respectively. The rate of technical errors for the *EGFR* scheme diminished from 25.2% to 1.5% between 2013–2014 and increased again to 5.2% in 2015 (Figure 2A). A great variety of methods was used for *EGFR* variant analysis over the years (Table 2). The use of laboratory-developed methods was gradually replaced by the implementation of NGS techniques.

**Table 2:** Overview of the used methods for *EGFR*, *ALK* and *ROS1* analysis in the external quality assessment schemes between 2012 and 2015

Scheme year	2012	2013	2014	2015
Method for *EGFR* variant analysis	NA	N = 107	N = 144	N = 114
Commercial, non-NGS based	NA	47 (43.9%)	95 (88.8%)	60 (56.1%)
*With FP and FN*	*NA*	*18 (16.8%)*	*56 (52.3%)*	*18 (16.8%)*
Laboratory developed, non-NGS based	NA	44 (41.1%)	42 (39.3%)	22 (20.6%)
*With FP and FN*	*NA*	*13 (12.1%)*	*24 (22.4%)*	*11 (10.3%)*
NGS-based	NA	1 (0.9%)	5 (4.7%)	33 (30.8%)
*With FP and FN*	*NA*	*0 (0.0%)*	*2 (1.9%)*	*7 (6.5%)*
Combination	NA	13 (12.1%)	2 (1.9%)	1 (0.9%)
*With FP and FN*	*NA*	*4 (3.7 %)*	*2 (1.9%)*	*0 (0.0%)*
Unspecified	NA	2 (1.9%)	0 (0.0%)	0 (0.0%)
*With FP and FN*	*NA*	*1 (0.9%)*	*0 (0.0%)*	*0 (0.0%)*
Total evaluated	NA	107 (100.0%)	144 (100.0%)	114 (100.0%)
*Total with FP and FN*	*NA*	*36 (33.6%)*	*84 (58.3%)*	*36 (31.6%)*

Antibody for ALK IHC	N = 29^**^	N = 48^**^	N = 96	N = 95
1A4	0 (0.0%)	0 (0.0%)	1 (1.0%)	4 (4.2%)
*With FP and FN*	*0 (0.0%)*	*0 (0.0%)*	*0 (0.0%)*	*1 (1.1%)*
4C5B8	0 (0.0%)	1 (2.0%)	0 (0.0%)	0 (0.0%)
*With FP and FN*	*0 (0.0%)*	*1 (2.0%)*	*0 (0.0%)*	*0 (0.0%)*
5A4	14 (46.7%)	18 (36.7%)	31 (32.3%)	35 (36.8%)
*With FP and FN*	*5 (16.7%)*	*3 (6.1%)*	*11 (11.5%)*	*1 (1.1%)*
ALK1	4 (13.3%)	3 (6.1%)	7 (7.3%)	4 (4.2%)
*With FP and FN*	*3 (10.0%)*	*2 (4.1%)*	*3 (3.1%)*	*0 (0.0%)*
D5F3	3 (10.0%)	26 (53.1%)	57 (59.4%)	52 (54.7%)
*With FP and FN*	*2 (6.7%)*	*13 (26.5%)*	*15 (15.6%)*	*3 (3.2%)*
SP8	2 (6.7%)	1 (2.0%)	0 (0.0%)	0 (0.0%)
*With FP and FN*	*2 (6.7%)*	*1 (2.0%)*	*0 (0.0%)*	*0 (0.0%)*
Unspecified	7 (23.3%)	0 (0.0%)	0 (0.0%)	0 (0.0%)
*With FP and FN*	*3 (10.0%)*	*0 (0.0%)*	*0 (0.0%)*	*0 (0.0%)*
Total evaluated	30 (100%)	49 (100%)	96 (100.0%)	95 (100.0%)
*Total with FP and FN*	*15 (50.0%)*	*20 (40.8%)*	*29 (30.2%)*	*5 (5.3%)*

Method/kit used for *ALK* FISH	N = 54^*^	N = 100^*^	N = 116	N = 111
Vysis ALK break apart FISH probe (Abbott)	41 (68.3%)	75 (72.8%)	79 (68.1%)	66 (59.5%)
*With FP and FN*	11 (18.6%)	16 (15.5%)	13 (11.2%)	8 (7.2%)
ALK LPS 019 breakapart probe (Cytocell)	0 (0.0%)	0 (0.0%)	1 (0.9%)	2 (1.8%)
*With FP and FN*	*0 (0.0%)*	*0 (0.0%)*	*1 (0.9%)*	*0 (0.0%)*

Method/kit used for *ALK* FISH	N = 54^*^	N = 100^*^	N = 116	N = 111
ALK FISH DNA Probe, Split Signal (Dako)	8 (13.3%)	10 (9.7%)	11 (9.5%)	13 (11.7%)
*With FP and FN*	*3 (5.1%)*	*0 (0.0%)*	*2 (1.7%)*	*1 (0.9%)*
Repeat-Free Poseidon ALK (2p23) Break Probe (Kreatech Diagnostics)	1 (1.7%)	3 (2.9%)	4 (3.4%)	3 (2.7%)
*With FP and FN*	*1 (1.7%)*	*0 (0.0%)*	*2 (1.7%)*	*0 (0.0%)*
Repeat-Free Poseidon ALK/EML4 t(2;2) inv(2) Fusion Probe (Kreatech Diagnostics)	4 (6.7%)	1 (1.0%)	0 (0.0%)	0 (0.0%)
*With FP and FN*	*0 (0.0%)*	*1 (1.0%)*	*0 (0.0%)*	*0 (0.0%)*
Master Diagnostica (not further specified)	0 (0.0%)	1 (1.0%)	0 (0.0%)	0 (0.0%)
*With FP and FN*	*0 (0.0%)*	*0 (0.0%)*	*0 (0.0%)*	*0 (0.0%)*
XT ALK Break Apart Probe (MetaSystems)	0 (0.0%)	0 (0.0%)	0 (0.0%)	1 (0.9%)
*With FP and FN*	*0 (0.0%)*	*0 (0.0%)*	*0 (0.0%)*	*0 (0.0%)*
Zyto*Light* SPEC ALK Dual Color Break Apart Probe (ZytoVision)	0 (0.0%)	1 (1.0%)	6 (5.2%)	14 (12.6%)
*With FP and FN*	*0 (0.0%)*	*0 (0.0%)*	*1 (0.9%)*	*0 (0.0%)*
Zyto*Light* SPEC ALK/EML4 TriCheck Probe (ZytoVision)	5 (8.3%)	7 (6.8%)	15 (12.9%)	12 (10.8%)
*With FP and FN*	*1 (1.7%)*	*3 (2.9%)*	*5 (4.3%)*	*0 (0.0%)*
Zytomed (not further specified)	0 (0.0%)	2 (2.0%)	0 (0.0%)	0 (0.0%)
*With FP and FN*	*0 (0.0%)*	*1 (1.0%)*	*0 (0.0%)*	*0 (0.0%)*
Unspecified	1 (1.7%)	3 (2.9%)	0 (0.0%)	0 (0.0%)
*With FP and FN*	*1 (1.7%)*	*0 (0.0%)*	*0 (0.0%)*	*0 (0.0%)*
Total evaluated	60 (100.0%)	103 (100.0%)	116 (100.0%)	111 (100.0%)
*Total with FP and FN*	*17 (28.3%)*	*21 (20.4%)*	*24 (20.7%)*	*9 (8.1%)*

Antibody ROS1 IHC	NA	NA	N = 31	N = 31
D4D6	NA	NA	31 (100.0%)	31 (100.0%)
*With FP and FN*	*NA*	*NA*	*7 (22.6%)*	*17 (54.8%)*

Methods/kit used for *ROS1* FISH			N = 56^*^	N = 68
Vysis 6q22 ROS1 Break Apart FISH Probe (Abbott)	NA	NA	4 (7.0%)	6 (8.8%)
*With FP and FN*	*NA*	*NA*	*1 (1.8%)*	*1 (1.5%)*
Vysis LSI ROS1 (Tel) Spectrum Orange Probe (Abbott); Vysis LSI ROS1 (Cen) Spectrum Green Probe (Abbott)	NA	NA	2 (3.5%)	3 (4.4%)
*With FP and FN*	*NA*	*NA*	*0 (0.0%)*	*0 (0.0%)*

Methods/kit used for *ROS1* FISH			N = 56^*^	N = 68
Vysis LSI ROS1 (Tel) Spectrum Orange Probe (Abbott)	NA	NA	9 (15.8%)	9 (13.2%)
*With FP and FN*	*NA*	*NA*	*1 (1.8%)*	*5 (7.4%)*
ROS1 breakapart Probe (Cytocell)	NA	NA	3 (5.3%)	4 (5.9%)
*With FP and FN*	*NA*	*NA*	*0 (0.0%)*	*1 (1.5%)*
SureFISH ROS1 break apart (Dako)	NA	NA	2 (3.5%)	4 (5.9%)
*With FP and FN*	*NA*	*NA*	*0 (0.0%)*	*1 (1.5%)*
REPEAT-FREE POSEIDON ROS1 (6q22) Break FISH Probe (Kreatech Diagnostics)	NA	NA	12 (21.1%)	9 (13.2%)
*With FP and FN*	*NA*	*NA*	*0 (0.0%)*	*3 (4.4%)*
6q22 (ROS1) DNA split FISH Probe (Medimiks)	NA	NA	0 (0.0%)	1 (1.5%)
*With FP and FN*	*NA*	*NA*	*0 (0.0%)*	*0 (0.0%)*
ZytoLight SPEC ROS1 - not specified (ZytoVision)	NA	NA	0 (0.0%)	1 (1.5%)
*With FP and FN*	*NA*	*NA*	*0 (0.0%)*	*1 (1.5%)*
ZytoLight SPEC ROS1 Dual Color Break Apart Probe (ZytoVision)	NA	NA	23 (40.4%)	30 (44.4%)
*With FP and FN*	*NA*	*NA*	*2 (3.5%)*	*2 (2.9%)*
ZytoLight SPEC ROS1/CEN 6 Dual Color Probe (ZytoVision)	NA	NA	1 (1.8%)	1 (1.5%)
*With FP and FN*	*NA*	*NA*	*1 (1.8%)*	*0 (0.0%)*
Unspecified	NA	NA	1 (1.8%)	0 (0.0%)
*With FP and FN*	*NA*	*NA*	*1 (1.8%)*	*0 (0.0%)*
Total evaluated	NA	NA	60 (100.0%)	68 (100.0%)
*Total with FP and FN*	*NA*	*NA*	*5 (8.8%)*	*14 (20.6%)*

For *ALK* and *ROS1* FISH, only laboratories that tested the regular FISH slides were taken into account, digital images were not considered. ^*^ Laboratories could enter two kits in the results form. ^**^ One Laboratory evaluated two antibodies and was counted twice. Abbreviations: *ALK*, anaplastic lymphoma kinase; *EGFR*, epidermal growth factor receptor; FP, false-positive; FN, false-negative; FISH, fluorescence *in situ* hybridization; IHC, immunohistochemistry; NA, not applicable because no EQA scheme was organized in this year; N, number of laboratories; NGS, next-generation sequencing; *ROS1*, proto-oncogene 1 tyrosine-protein kinase.

For *ALK* analysis, there was a decrease in error rates between 2012 and 2015 for the three subschemes. For FISH digital, error rates decreased from 3.6% towards 0.4% over time. For FISH and IHC on the tissue samples, error rates diminished from 7.3% to 2.1% and from 13.0% to 1.3%, respectively. Error rates for IHC were higher compared to FISH in 2014, and compared to FISH digital analysis between 2012 and 2014 (Figure 1B). The percentage of technical failures remained stable for *ALK* FISH digital analysis, but decreased for *ALK* FISH from 10.0% to 5.6%. For ALK IHC, a transient rise of 6.3% technical failures was observed in 2013.

For ALK IHC, a shift in the primary antibody clone was observed from 5A4 (60.9%) in 2012 towards D5F3 (54.7%) in 2015 (Table 2). In 2015, 73 laboratories from 25 countries submitted their stained IHC slides for the technical microscopic assessment. Their average technical score was 76.6%, or 3.83 on 5 (data not shown). A successful score of more than or equal to 4 out of 5 points was awarded to 48/73 (65.8%) laboratories. Seven out of 73 participants (9.6%) were not successful with a score of 2 or less on a total of 5 points. The borderline score of 3/5 was awarded to 18 out of 73 (24.7%) laboratories. No correlation was observed of the staining score with a particular primary antibody clone, its dilution, incubation time, or the applied detection system.

For *ALK* FISH, the Vysis ALK break apart FISH probe (Abbott) was used by the majority of laboratories during all scheme years (Table 2). The evaluation of at least 50 neoplastic nuclei for *ALK* FISH and FISH digital improved across the subsequent EQA scheme years (Table 3). The percentage of samples in which less than 50 nuclei were counted was higher for the digital FISH cases as compared to FISH. No correlation was observed between the number of nuclei counted and the scheme error rate. For the negative cases, a distinction was made between counting less than 50 or less than 43 nuclei, as counting less than 43 nuclei could result in a change of outcome when the unevaluated 7 nuclei are positive, eventually exceeding the 15% cutoff for positivity.

**Table 3:** Fraction of samples (%) for which less than 50 nuclei were enumerated by the participants of the FISH and FISH digital subschemes for *ALK* and *ROS1*, and the fraction (%) of them including false-positive, false-negative or technical failures

Subscheme	*ALK* FISH				*ALK* FISH digital				*ROS1* FISH		*ROS1* FISH digital
Scheme year	2012	2013	2014	2015	2012	2013	2014	2015	2014	2015	2015
**<50 nuclei evaluated in a positive case**	**46/161 (28.6%)**	**13/208 (6.3%)**	**21/232 (9.1%)**	**8/222 (3.6%)**	**34/130 (26.2%)**	**22/212 (10.4%)**	**15/162 (9.3%)**	**18/222 (8.1%)**	**6/56 (10.7%)**	**6/136 (4.4%)**	**4/68 (5.9%)**
Nr of FN	5/46 (10.9%)	1/13 (7.69%)	2/21 (9.5%)	0/8 (0.0%)	3/34 (8.8%)	19/22 (86.4%)	0/15 (0.0%)	0/18 (0.0%)	0/6 (0.0%)	0/6 (0.0%)	0/4 (0.0%)
Nr of technical failures	18/46 (39.1%)	0/13 (0.0%)	11/21 (52.4%)	5/8 (62.5%)	7/34 (20.6%)	3/22 (13.6%)	2/15 (13.3%)	7/18 (38.9%)	3/6 (50.0%)	4/6 (66.7%)	1/4 (25.0%)
**43<50 nuclei evaluated in a negative case**	**3/108 (2.8%)**	**7/312 (2.2%)**	**14/696 (2.1%)**	**1/333 (0.3%)**	**10/130 (7.7%)**	**10/212 (4.7%)**	**8/162 (4.9%)**	**4/333 (1.2%)**	**2/392 (0.5%)**	**1/204 (0.5%)**	**3/204 (1.5%)**
Nr of FP	0/3 (0.0%)	6/7 (85.7%)	0/14 (0.0%)	0/1 (0.0%)	1/10 (10.0%)	10/10 (100.0%)	0/8 (0.0%)	0/4 (0.0%)	0/2 (0.0%)	1/1 (100.0%)	0/3 (0.0%)
Nr of technical failures	0/3 (0.0%)	0/7 (0.0%)	0/14 (0.0%)	0/1 (0.0%)	0/10 (0.0%)	0/10 (0.0%)	0/8 (0.0%)	0/4 (0.0%)	0/2 (0.0%)	0/1 (0.0%)	0/3 (0.0%)
**<43 nuclei evaluated in a negative case**	**21/108 (19.4%)**	**11/312 (3.5%)**	**60/696 (8.6%)**	**26/333 (7.8%)**	**20/130 (15.4%)**	**22/212 (10.4%)**	**10/162 (6.2%)**	**11/333 (3.3%)**	**68/392 (17.3%)**	**8/204 (3.9%)**	**6/204 (2.9%)**
Nr of FP	1/21 (4.8%)	11/11 (100.0%)	0/60 (0.0%)	0/26 (0.0%)	0/20 (0.0%)	18/22 (81.8%)	0/10 (0.0%)	0/11 (0.0%)	1/68 (1.5%)	0/8 (0.0%)	0/6 (0.0%)
Nr of technical failures	5/21 (23.8%)	0/11 (0.0%)	44/60 (73.3%)	23/26 (88.5%)	0/20 (0.0%)	4/22 (18.2%)	3/10 (30.3%)	5/11 (45.5%)	54/68 (79.4%)	8/8 (100.0%)	1/6 (16.7%)

Educational cases are not included in the total sample number. For the negative cases, a distinction was made between counting less than 50 or even less than 43 nuclei, since counting less than 43 nuclei could result in a change of outcome when the unevaluated 7 nuclei are positive, eventually exceeding the 15% cutoff for positivity. Abbreviations: *ALK*, anaplastic lymphoma kinase; FISH, fluorescence *in situ* hybridization; *ROS1*, proto-oncogene 1 tyrosine-protein kinase; FP, false-positive; FN, false–negative; Nr, Number

In contrast to *EGFR* and *ALK* analysis, error rates for *ROS1* analysis increased between 2014 and 2015. For FISH and IHC the rates increased from 1.1% to 4.3% and 4.3% to 13.6%, respectively (Figure 1C). The *ROS1* FISH digital cases were introduced in 2015, and an error rate of 3.2% was observed. Similar to *ALK* analysis, error rates were higher for ROS1 IHC, compared to *ROS1* FISH in 2014 and 2015 and *ROS1* FISH digital in 2015. In contrast to the error rates, technical failures for *ROS1* subschemes diminished between the two EQA rounds, and were higher for FISH and FISH digital analysis as compared to IHC (Figure 2C).

In 2014–2015, the D4D6 primary antibody clone was the only one routinely used for the detection of *ROS1* expression (Table 2). In spite of the same clone, the number of laboratories obtaining a false-positive or false-negative result increased from 7/31 (22.6%) towards 17/31 (54.8%) (data not shown). For FISH, the ZytoLight SPEC ROS1 Dual Color Break Apart Probe (ZytoVision) was the most popular for *ROS1* rearrangement analysis during both EQA scheme years (Table 2). Similar to the *ALK* scheme, the evaluation of at least 50 neoplastic nuclei for FISH and FISH digital improved across the subsequent EQA schemes (Table 3), although more nuclei were counted in the FISH digital images than in FISH samples.

## DISCUSSION

Since 2012, ESP EQA schemes have been organized to assess and improve the current state of biomarker testing in NSCLC. In 2016, *ROS1* analysis has been added as a requirement to the label of crizotinib [5, 6], which called to re-evaluate the performance of laboratories during the four EQA scheme years.

The EQA results demonstrate that individual laboratories improve their testing quality if they participate to more subsequent EQA scheme years. (Table 1). In addition, graphs display a general decrease in error rates for *EGFR* and *ALK* analysis and an increase in errors for *ROS1* analysis (Figure 1), across scheme years. These findings suggest that the feedback provided at the end of an EQA scheme have positively influenced the participants’ test performance. However, increased experience can also be an improving factor, although difficult to quantify as routine diagnostics is a continuously developing field parallel to EQA participation. Indeed, further research is needed to investigate the underlying causes of laboratory errors, and how sample complexity can contribute to these. However, the high number of participants allows us to reliably estimate the average error rate. While other EQA results in NSCLC have been reported [21–23, 25], unique to our data is the longitudinal comparison between scheme years, markers and techniques.

Despite the improvement of *EGFR* variant analysis over the years (Figure 1A), the remaining high error rates in 2015 were mostly due by the misidentification of c.2369C>T p.(Thr790Met), although unrelated to the technique used. Between 2013–2015, five cases (two resections and three cell-lines) including this variant were included, and more errors were observed in the resection specimens compared to cell-lines. As this variant usually occurs at low frequencies in routine, and allele frequencies varied between 10%-50%, the exact reason why this variant seems to be difficult remains to be elucidated. Previously, a high percentage of false-negative results was also observed for cell-lines with this variant in three consecutive EQA scheme years in France [24]. Reliable detection of c.2369C>T p.(Thr790Met) is clinically important as it is present in 50% of treated *EGFR*-mutated tumors, and confers resistance to first and second generation TKI’s [28]. Moreover, the FDA and EMA approved osimertinib as a third generation TKI for the treatment of progressive NSCLC containing this variant, in 2015 and 2016 respectively [29, 30]. Additional awareness is thus needed, and EQA providers should routinely include challenging samples in EQA schemes, if necessary by the inclusion of cell lines if resection material is limited available. Moreover, for *EGFR* analysis a large inter-observer variability in the estimation of the percentage of neoplastic cells in the samples was observed. Pictures of the tissue area selected for DNA extraction that were optionally provided by some of the participants, reveal that the marked area differs among participants. This variability is well-known in molecular pathology, and additional research is required to evaluate the different methods for neoplastic cell estimation and their influence on the performance score.

For *ALK* analysis, error rates displayed an obvious improvement for all subschemes and years. Error rates were higher for *ALK* IHC analysis, compared to the FISH and the FISH digital subscheme, in line with previously reported EQA results in 2012 and 2013 [17]. Immunohistochemistry is frequently used as a screening tool prior to FISH confirmation, and although some studies describe an equal sensitivity, some discrepancies between both techniques have been reported also [31]. For *ALK* IHC, a shift occurred from the 5A4 towards the D5F3 antibody clone as the D5F3 CDx IHC assay (Ventana) was approved by the FDA in the USA and China since 2015, and pathologists were provided with a guide for the interpretation of a binary score for this antibody clone. In addition, several studies [32–34] revealed a similar sensitivity (96-100%) of both the 5A4 and D5F3 clones for ALK IHC staining.

During the technical assessment for ALK IHC, no correlation was observed between the staining quality or the ALK IHC performance and the used antibody clone, the process of epitope retrieval, incubation time and temperature, or detection system. This is in contrast to recently published EQA results from the United Kingdom National External Quality Assessment Service [35], for which a worse performance was related to specific detection methods. However, the included sample types in the ESP EQA scheme and distribution of methodologies differ, so longitudinal data of the next technical evaluations are necessary to provide insight into recurrent difficulties for ALK IHC.

For *ALK* FISH analysis, error rates were higher for FISH as for the interpretation of the digital images. This can be explained by the fact that FISH encompasses additional factors related to sample quality and assay execution, making this technique more error prone compared to interpretation only. Besides the reduced error rate, digital FISH also showed a decreased variation in the percentage of observed positive signals as compared to FISH (data not shown), suggesting that a factor of tissue heterogeneity might have been involved. As for IHC, the most frequently used methodology is the VYSIS ALK Break Apart FISH Probe Kit (Abbott) which is the FDA approved test to determine *ALK* status. In addition, it is recommended to enumerate at least 50 nuclei before considering a FISH or FISH digital case as either positive or negative [18–20]. Our results demonstrate an improvement in enumeration practices across scheme years (Table 3), in line with the observations of 2012 and 2013 [17]. Surprisingly, in contrast to the recommendations, participants evaluating less than 50 nuclei in a positive case or less than 43 nuclei in a negative case did not perform worse in the EQA schemes. This could be due to the fact that equivocal cases are denoted as educational. Counting less nuclei is especially striking for the digital images as they were preselected and validated to contain at least 50 evaluable nuclei, and all participants receive access to identical cases. However, digital FISH images might not follow the same laboratory course as the regular slides [17], hence the difference for *ROS1* testing. Error rates increased between 2014 and 2015 for both FISH and IHC (Figure 1C). First of all, this is explained by the fact that *ROS1* was not yet a routinely used predictive biomarker in 2014–2015, and participants might still be in the learning/validation phase of the analysis. In 2013, a similar increased error rate was also reported after the inclusion of *NRAS* to the label of cetuximab and panitumumab for treatment of patients with metastatic colorectal cancer [13]. Also, guidelines did not or only briefly mention *ROS1* specific recommendations until 2016 [36–37]. Secondly, in 2014 four of ten cases consisted of cell-lines, whereas in 2015 all five distributed cases were resection specimens, suggesting that tissue heterogeneity could play a role. To ensure the adequacy of the resection material, the ESP selects clinical samples containing at least 30% neoplastic cells, and provides H&E stained slides to the participants.

Similar to the *ALK* scheme, error rates were higher for ROS1 IHC as compared to FISH or FISH digital analysis. First of all, in 2015 one particular ROS1 positive case has led to an increased error rate for IHC, as 15 of 31 laboratories reported a false-negative result. Although a consensus score of at least 75% was not reached, the case was not denoted as educational based on the absence of problems during validation, the adequate FISH results and re-evaluation by the assessors. Leaving out this sample, an error rate of 4.8% instead of 13.6% could be observed (Figure 1C, Black triangle). Nevertheless, with recent changes in the status of crizotinib, *ROS1* test requests are expected to increase over time and quality of the test results must be assured.

Focusing on the technical failures, the increase for *EGFR* analysis could be explained by an increase of NGS users between 2014 and 2015 from 4.2% to 28.9% (Table 2). Although in 2015 less genotyping errors were made by the NGS users (χ2 test, p=0.129), the percentage of laboratories with technical errors was higher for this group with 30.3% (n=33) as compared to non-NGS users with 23.5% (n=81), while not significant (χ2 test, p=0.447). Previously, the French EQA results also revealed increased error rates over time, among other things due to the rising number of genes to be analyzed in parallel [24]. For *ALK* and *ROS1*, technical failures were less abundant for IHC, compared to the FISH digital and FISH subscheme. This could be explained by four reasons: (1) the fact that although interpretation errors might occur, IHC is a more routinely used method with automated steps as compared to FISH. (2) In 2013, a tissue micro-array was provided for ALK IHC and many participants indicated a loss of one of the six provided cores which was subsequently considered as a technical failure. (3) While several cases were considered educational for *ALK* FISH, this was not the case for ALK IHC (Table 4), resulting in an elevated number of technical failures for this subscheme. (4) For FISH digital analysis the distance between signals may be difficult to estimate or may be overlapping in the vertical axis, which could also result in a higher number of participants who denote the sample as not evaluable as compared to FISH analysis [31]. However, all participants received access to identical FISH digital images, and cases were included if at least 75% of participants were able to correctly them.

**Table 4:** Overview of the distributed samples for each subscheme in the ESP lung EQA schemes between 2012-2015

Subscheme	Scheme year	Nr of participants	Nr of samples distributed	Sample origin	Number of slides distributed per sample	Section thickness (μm)	Samples included in performance score	Educational samples (not included in performance score)
*EGFR* analysis	2013	107	4	4 cell lines	1	5	1 WT, 3 mut	0 WT, 0 mut
	2014	144	9	5 cell lines, 4 resections	2	5	2 WT, 7 mut	0 WT, 0 mut
	2015	114	10	10 resections	2	5	5 WT, 4 mut	0 WT, 1 mut
ALK IHC	2012	29	8	4 cell lines, 4 resections	2	5	4 +ve, 4 -ve	0 +ve, 0 -ve
	2013	48	12	6 cell lines, 6 resections	1	5	3 +ve, 9 -ve	0 +ve, 0 -ve
	2014	96	10	4 cell lines, 6 resections	2	3	2 +ve, 7 -ve	1 +ve, 0 -ve
	2015	95	5	5 resections	2	3	2 +ve, 3 -ve	0 +ve, 0 -ve
*ALK* FISH	2012	54	8	4 cell lines, 4 resections	2	5	3 +ve, 2 -ve	1 +ve, 2 -ve
	2013	100	12	6 cell lines, 4 resections	1	5	2 +ve, 3 -ve	2 +ve, 5 -ve
	2014	116	10	4 cell lines, 6 resections	1	5	2 +ve, 6 -ve	1 +ve, 1 -ve
	2015	111	5	5 resections	1	5	2 +ve, 3 -ve	0 +ve, 0 -ve
*ALK* FISH digital	2012	65	4	Digital images from resection specimens	NA	NA	2 +ve, 2 -ve	0 +ve, 0 -ve
	2013	106	4				2 +ve, 2 -ve	0 +ve, 0 -ve
	2014	81	4				2 +ve, 2 -ve	0 +ve, 0 -ve
	2015	111	5				2 +ve, 3 -ve	0 +ve, 0 -ve
ROS1 IHC	2014	31	10	4 cell lines, 6 resections	2	3	2 +ve, 8 -ve	0 +ve, 0 -ve
	2015	31	6	5 resections	2	3	2 +ve, 3 -ve	1 +ve, 0 -ve
*ROS1* FISH	2014	56	10	4 cell lines, 6 resections	2	5	1 +ve, 7 -ve	1 +ve, 1 -ve
	2015	68	6	1 cell line, 5 resections	1	5	2 +ve, 3 -ve	1 +ve, 0 -ve
*ROS1* FISH digital	2015	68	5	Digital images from resection specimens	NA	NA	1 +ve, 3 -ve	1 +ve, 0 -ve

The schemes of 2012 and 2013 are two pilot EQA schemes for which the set-up has previously been reported [17]. The following variants were part of the *EGFR* variant analysis schemes: c.2155G>A p.(Gly719Ser), c.2156G>C p.(Gly719Ala), c.2235_2249del p.(Glu746_Ala750del), c.2327G>A p.(Arg776His), c.2369C>T p.(Thr790Met), c.2573T>G p.(Leu858Arg), c.2300_2308dup p.(Ala767_Val769dup). Abbreviations: *ALK*, anaplastic lymphoma kinase; *EGFR*, epidermal growth factor receptor; EQA, external quality assessment; FISH, fluorescence *in situ* hybridization; IHC, immunohistochemistry; mut, mutated; NA, not applicable; Nr, Number; *ROS1*, proto-oncogene 1 tyrosine-protein kinase; WT, wild-type; +ve, positive; -ve, negative.

To conclude, results of this study illustrate that EQA helps to uncover problems in a timely manner. Importantly, this study also highlights that on an individual laboratory level, more participations are correlated with an improved performance of biomarker analysis in NSCLC. Although, the exact mechanism might be difficult to elucidate as molecular pathology is a field of continuous evolvement and gained experience. Room for improvement is still apparent, especially for ROS1 IHC or the detection of low frequency resistance mutations like c.2369C>T p.(Thr790Met). A large variety was also observed in the estimation of the neoplastic cell content and in the different analysis methods.

To improve error rates for biomarker analysis, frequent EQA participation is essential to strive to high quality biomarker analyses irrespective of the used methodologies to ensure patient safety at all times.

In addition, future research needs to be performed to get more insight into the exact causes of the error rates linked to specific variants/methodologies, and how sample characteristics and the different ways of neoplastic cell estimation contribute to these problems. The yearly organization of these EQA schemes provides an essential platform to evaluate these topics in a longitudinal manner, and based on the findings reported in this manuscript, projects are currently being set-up. Ultimately these will result in more detailed laboratory guidelines to overcome specific or systematic challenges.

## MATERIALS AND METHODS

The set-up of each ESP Lung EQA scheme was determined beforehand by a steering committee of international experts in molecular diagnostics, according to the guideline on the requirements of external quality assessment programs in molecular pathology [14]. The organization by the coordination center was performed in accordance with the ISO 17043 standard for conformity assessment of proficiency testing [26]. Formalin-fixed paraffin-embedded (FFPE) samples were collected and the experts made a final selection to be provided to the participants. A minimum of three reference laboratories assessed the adequacy of each sample using their routinely applied detection methods.

Participants were able to register, submit data and access their results via a password-protected central database, accessible through the ESP Lung EQA scheme website (http://lung.eqascheme.org). Registration was open to all laboratories world-wide and allowed to select different subschemes depending on the marker (*EGFR*, *ALK* and/or *ROS1*) and method (variant analysis, FISH and/or IHC) of interest. An overview of the samples distributed during each subscheme is represented in Table 4.

Between 2012 and 2014, participants of the FISH subscheme could optionally interpret five additional digital FISH images, which was made mandatory in 2015. These digital cases were created with the Vysis ALK Break Apart FISH Probe kit (Abbott) for *ALK* and the Vysys 6q22 ROS1 Break Apart FISH probe RUO kit (Abbott) for *ROS1*, and captured images were validated by the reference laboratories. The images were made available online, along with the digitized hematoxylin and eosin stained slides for pathologist review of the FFPE material. Access was provided via the ESP Lung EQA website and the PathXL platform (PathXL, Belfast, Northern Ireland; http://www.pathxl.com/). In 2015, the ALK IHC subscheme was expanded by a technical microscopic assessment of the slides’ immunohistochemical staining by a team of pathologists.

Participants were asked to analyze the samples using their routine testing procedures. In case of *EGFR* analysis, an estimation of the percentage of neoplastic cells in the tissue should be given. For *ALK*/*ROS1* FISH, participants were asked to evaluate at least 50 nuclei and to apply a 15% threshold for positivity as recommended [19]. To reflect clinical practice, the deadline for submission of the results was 14 calendar days after sample receipt [27]. Additional information regarding laboratory characteristics and the applied methods was requested via the online submission system on their account.

The scheme results were evaluated by a team of international assessors for each individual subscheme in agreement with the guidelines on the requirements of EQA programs in molecular pathology [20]. Samples for which more than 25% of participants were not able to obtain conclusive results were considered educational and were excluded from the performance score. For other samples, laboratories could obtain a maximum of two points for a correct outcome. During the technical assessment, a general technical score on five points was provided for the five slides provided in the scheme. Successful participation was defined as all laboratories with equal to or more than four on five. A score of three was considered borderline. Laboratories could access their results and individual feedback from current and previous EQA schemes via their account.

Error rates for a subscheme were calculated by summating the total number of false-positive and false-negative results or wrong variants, divided by the total number of samples for which a result has been obtained by all participants of the subscheme, and were presented in a descriptive manner on sample level. For the FISH and FISH digital subschemes, only samples for which more than or equal to 50 nuclei were counted were included. The rate of technical failures was determined by dividing the total number of samples for which a laboratory reported a not-contributive result, by the total number of samples analyzed. For every scheme year between 2012 and 2015, the error and technical failure rates are represented per marker (*ALK, ROS1, EGFR*) and method of analysis (variant analysis, FISH, IHC). To evaluate the improvement of the individual laboratories upon participation to EQA, their average genotyping score was calculated, after participation to only one, two, three or four subsequent EQA schemes.

Graphs were created using GraphPad Prism version 7.00 (GraphPad Software Inc, La Jolla, CA, USA). The tables representing the methods used by the participants to analyze the samples are displayed as reported by the participants.

### The EQA assessors expert group

Bubendorf Lukas – University Hospital Basel, Switzerland

Cabillic Florian – Cytogenetics and Cellular Biology Department, CHU de Rennes, INSERM, INRA, Univ Rennes 1, Univ Bretagne Loire, Nutrition Metabolisms and Cancer, Rennes, France

’t Hart Nils - University Medical Center Groningen, Groningen, The Netherlands

Delen Sofie – KU Leuven, KU Leuven, Biomedical Quality Assurance Research Unit, Leuven, Belgium

Dequeker Elisabeth – KU Leuven, Biomedical Quality Assurance Research Unit, University Hospital UZ Leuven, Leuven, Belgium

Keppens Cleo - KU Leuven, Biomedical Quality Assurance Research Unit, Leuven, Belgium

Miller Keith – UKNEQAS, London, United Kingdom

Pauwels Patrick – University Hospital Antwerp, Edegem, Belgium

Ryska Ales – Charles University Medical Faculty Hospital, Hradec Kralove, Czech Republic

Schuuring Ed – University Medical Center Groningen, Groningen, The Netherlands

Tack Véronique – KU Leuven, Biomedical Quality Assurance Research Unit, Belgium

Tembuyser Lien – KU Leuven, Biomedical Quality Assurance Research Unit, Leuven, Belgium

Thunnissen Erik – VU University Medical Centre Amsterdam, Amsterdam, The Netherlands

Tornillo Luigi - University of Basel, Basel, Switzerland/GILAB AG, Allschwil, Switzerland

Warth Arne – University Hospital Heidelberg, Heidelberg, Germany

Weichert Wilko – Technical University Munich (TUM), Munich, Germany

Zwaenepoel Karen - University Hospital Antwerp, Edegem, Belgium

## Abbreviations

*ALK*: anaplastic lymphoma kinase
*EGFR*: epidermal growth factor receptor
ESP: European Society of Pathology
EQA: External Quality Assessment
FFPE: formalin-fixed paraffin embedded
FISH: fluorescence *in situ* hybridization
IHC: immunohistochemistry
NGS: next-generation sequencing
*ROS1*: proto-oncogene 1 tyrosine-protein kinase
TKI: tyrosine kinase inhibitor
